# Systemic Factors Affecting Human Beta-Defensins in Oral Cavity

**DOI:** 10.3390/pathogens13080654

**Published:** 2024-08-02

**Authors:** Nur Atalay, Nur Balci, Mervi Gürsoy, Ulvi Kahraman Gürsoy

**Affiliations:** 1 Department of Periodontology, Institute of Dentistry, University of Turku, Lemminkäisenkatu 2, 20520 Turku, Finland; dtnuratalay@gmail.com (N.A.); mervi.gursoy@utu.fi (M.G.); 2 Department of Periodontology, Faculty of Dentistry, Medipol University, Goztepe Mahallesi, Ataturk Caddesi 40, Beykoz, 34815 Istanbul, Turkey; nbalci@medipol.edu.tr; 3 Welfare Division, Oral Health Care, 20540 Turku, Finland

**Keywords:** defensins, periodontium, periodontal diseases, retinoids, vitamin D, aging, pregnancy, hyperglycemia

## Abstract

Human beta-defensins are host defense peptides with broad antimicrobial and inflammatory functions. In the oral cavity, these peptides are produced mainly by the keratinocytes of the epithelium; however, fibroblasts, monocytes, and macrophages also contribute to oral human beta-defensin expressions. The resident and immune cells of the oral cavity come into contact with various microbe-associated molecular patterns continuously and simultaneously. The overall antimicrobial cellular response is highly influenced by local and environmental factors. Recent studies have produced evidence showing that not only systemic chronic diseases but also systemic factors like hyperglycemia, pregnancy, the long-term use of certain vitamins, and aging can modulate oral cellular antimicrobial responses against microbial challenges. Therefore, the aim of this narrative review is to discuss the role of systemic factors on oral human beta-defensin expressions.

## 1. Introduction

Humans are holobionts. The human body is inhabited by hundreds of different microbial species living in their own ecosystems, which are developed on various organs, such as on the skin, gut, respiratory tract, or oral cavity. The interaction between the human body and microorganisms is mainly symbiotic, meaning that these microorganisms contribute to various metabolic functions and immune response regulations. While a majority of the microbial species that inhabit a healthy oral cavity are accepted as commensal, some species may act as opportunistic pathogens, especially in individuals with an increased susceptibility to chronic diseases [1]. This, indeed, makes a clear-cut definition of commensal, opportunistic, and pathogenic microorganisms difficult. In the oral cavity, the epithelium serves as the first physical barrier against a continuous microbial challenge. The shedding and high turnover rate of the oral gingival epithelium contribute to the mechanical barrier property of periodontal tissues against microbial invasions [2]. Moreover, to suppress microbial overgrowth, the oral epithelium secretes various antimicrobial agents, including human β-defensins (hBDs) [3]. The expression of these small cationic antimicrobial peptides is mainly regulated by the interactions between eukaryotic pattern-recognition receptors and microbe-associated molecular patterns; however, external factors, such as the long-term use of vitamins A and D, hyperglycemia, pregnancy, and aging, affect their expression and secretion profiles.

Antibiotics are first-choice adjuvant drugs in the treatment of oral infections; however, their use is highly restricted due to their side effects. Moreover, the development of new antibiotics is now in a dramatic decline, while infections due to multidrug-resistant bacteria are steadily increasing. One other strategy in preventive and therapeutic dentistry is to support immune mechanisms to boost the expression of the body’s own host defense peptides, defensins. While this strategy has succeeded in laboratory studies, no commercial product has come to market due to a lack of delivery formulations. An in-depth understanding of the contribution of lifestyle and systemic factors on host defense peptide expressions will help us (1) to eliminate the lifestyle-related risk factors that suppress host defense peptide expressions, especially in individuals that are prone to develop oral diseases and (2) to develop methods to boost the body’s own antimicrobial peptide expressions to enhance the defense against oral infections and inflammatory diseases.

The aim of this narrative review is to describe the expression profile of gingival hBDs in relation to systemic factors. While doing so, we will shortly describe (1) periodontal tissues, (2) infection-induced inflammatory diseases of the periodontium, and (3) hBD expression profiles in periodontal tissues in relation to periodontitis. The gene regulation of hBD expression, chemical structures of hBDs, and hBD levels in relation to systemic diseases are not in the scope of this review, and readers are advised to refer to well-written articles on these topics [4,5].

## 2. Periodontium

The periodontium is composed of tooth-supporting tissues, i.e., gingiva, periodontal ligament, cementum, and alveolar bone. The periodontium has a unique vascular structure, lymphatic system, and specialized innervation networks, providing the necessary support for teeth to perform their functions in the mouth [6]. Even though the components of periodontal tissues differ in terms of their tissue architecture, they also perform in harmony as a single entity.

The gingiva covers the alveolar mucosa and ends in a knife-edge shape in accordance with the contour of the teeth it surrounds [7]. The gingiva, which is formed of the epithelium (oral, sulcular, and junctional epithelium) and connective tissue (lamina propria), is anatomically divided into marginal, attached, and interdental regions. Although the epithelium is predominantly cellular in nature, the connective tissue contains fewer cells and consists mainly of collagen fibers and ground substances [8]. The gingival epithelium provides selective permeability in the oral environment with its physical structure, host defense peptides, and inflammatory cells [6]. The integrity of the epithelial barrier can be disrupted by a variety of microbial pathogens that attack cell–cell junctions or that stimulate cellular cytotoxicity. In addition to the barrier effect of the epithelium, the high regenerative rate of the cells in the gingival sulcus plays an important role in the maintenance of periodontal health [9]. The cellular regenerative rate, which varies between 4 and 6 days in junctional epithelial cells and 6 and 12 days in sulcular epithelial cells, is advantageous, as it allows for the rapid replacement of cells and tissue components damaged by bacterial invasions [10]. The gingival connective tissue is a fibrous connective tissue containing collagen fibers (60%), fibroblasts (5%), vessels, nerves, and extracellular matrices (35%) [11]. Connective tissue fibers make the gingiva resilient against chewing forces and maintain tissue integrity. The periodontal ligament, which lies between the alveolar bone and the cementum on the root surface, is a cell-rich fibrous connective tissue and contains vessels and nerves. Thanks to its ability to stretch up to one and a half times its volume, it absorbs the mechanical load during chewing and presents a regulatory role in proprioception. Its cellular components are formed of various tissue-forming (fibroblasts, endothelial cells, cementoblasts, and osteoblasts) and tissue-resorbing (osteoclasts) cells. Indeed, it has an important role in the tissue homeostasis and regeneration of the periodontium. The periodontal ligament matrix contains proteoglycans, glycoproteins, hyaluronic acid, glycolipids, minerals, and growth factors [12]. Cementum is the calcified avascular mesenchymal tissue that covers the root dentin, into which periodontal ligament fiber bundles are inserted. It accumulates in layers throughout life without undergoing resorption and remodeling [13]. Inorganic hydroxyapatite constitutes 45–50% of the cementum, and the remaining part consists of organic collagen tissues and non-collagenous matrix proteins. Some of the growth factors located in cementum enable its proliferation and differentiation. Indeed, these growth factors play an active role in the homeostasis and regeneration of the gingiva, periodontal ligament, and alveolar bone [11]. The alveolar bone is part of the jawbone that supports the teeth and forms the space called alveoli (tooth socket) that accommodate the roots of teeth. It contains vessels and nerves of the teeth and periodontium. Alveolar bone cells are osteoblasts, osteoclasts, osteocytes, and undifferentiated connective tissue cells [7]. The alveolar bone is the least stable among the periodontal tissues and undergoes a continuous remodeling process of construction and destruction in the face of external forces.

## 3. Periodontal Health and Diseases

Periodontal diseases are multifactorial chronic inflammatory diseases that can cause the destruction of periodontal tissues and even tooth loss as a result of the complex relationships between microbial dental plaque and the host [14]. The periodontium can be affected by various acquired and hereditary diseases/conditions. The transition process from periodontal health to disease and periodontal disease progression are managed by an immune response resulting from pathogenic microorganisms and the interaction of these microorganisms with the host [15].

Periodontal health is the state of not showing any clinical signs of periodontal inflammation on an anatomically intact or reduced periodontium [16]. Based on this general health framework, periodontal healthy individuals can include patients without gingivitis, periodontitis, or a disease associated with other periodontal conditions and with a history of a successfully treated periodontal disease. Furthermore, a reduced periodontium diagnosis is used for individuals who have no disease-related symptoms and no bleeding, edema, and redness on probing the gums, although a clinical attachment and bone loss are observed [17]. In addition, clinical periodontal health includes the physiological immune state, which encapsulates the balance of biological and inflammatory markers that ensure periodontal homeostasis [18]. 

Gingivitis initially develops in response to microbial dental plaque. Clinically, bleeding, redness, and edema are observed, and two early signs of gingival inflammation are bleeding after probing and an increased gingival crevicular fluid volume. In most cases, the cellular infiltrate at the bleeding site on probing is predominantly lymphocytes (characteristic in stage II or early gingivitis). Since the junctional epithelium is more permeable than the sulcular epithelium, neutrophils chemotactically migrate from the blood vessels in the connective tissue. The series of events that begin with an elevated chemokine (including hBDs) expression by epithelial cells and neutrophil migration lead to the early stages of gingivitis [19]. Studies conducted in recent years aim to elucidate possible biomarkers and disease mechanisms in the diagnosis of gingivitis. Wang et al. suggest that the disruption of the balance between Th17/Treg may be associated with the progression of gingivitis [20]. Additionally, it has been revealed that the levels of the Secretory leukocyte protease inhibitor (SLPI), which has broad-spectrum antimicrobial activity, increases significantly during gingivitis and periodontitis [21]. Different systemic factors, including elevated sex steroids (i.e., puberty and pregnancy), vitamin C deficiency, or nutritional deficiencies, also contribute to the development of gingivitis by modifying the host response [22].

Gingival inflammation reduces when oral hygiene is improved, and microbial dental plaque is eliminated. If left untreated, an apical migration of the connective tissue attachment may occur, and gingivitis may advance to periodontitis. For periodontitis to develop, gingivitis must first occur, but not all gingivitis turns into periodontitis [23]. Clinically, signs of periodontitis are generally a bluish-red gingiva, a thickening of the gingival margin, bleeding, suppuration, tooth mobility and diastema formation, the presence of deep periodontal pockets, and alveolar bone loss. Periodontitis is a dysbiotic disease when evaluated from a microbial perspective. Because the innate immune response against bacteria in the oral cavity plays a key role in periodontal defense, there is a constant “arms” race between the host and bacteria, even in clinically healthy periodontal tissues [24]. When microbes or their by-products (microbe-associated molecular patterns, MAMPs) interact with pattern-recognition receptors (PRRs), such as toll-like receptors (TLRs), of the host, inflammatory responses occur. Interactions of MAMPs with PRRs activate intracellular signaling mechanisms. With this activation, neutrophils constitute the first cellular host defense and target to eliminate pathogens by secreting granular proteins such as elastase, cathepsin G, myeloperoxidase, and peptidoglycan recognition proteins. However, while trying to clear pathogens in the tissue, their presence at high levels can also cause oxidative damage with an excessive production of reactive oxygen species [23]. A wide variety of cytokines and chemokines have been demonstrated to play roles in the pathogenesis of periodontitis to soft tissue destruction and bone resorption. Pro-inflammatory cytokines such as interleukin (IL)-1α, IL-1β, IL-6, IL-23, and IL-17 and tumor necrosis factor alpha (TNF-α) are held responsible, whereas interferon gamma (IFN-γ), IL-4, IL-1RA, IL-10, and IL-12 are reported as regulatory cytokines during periodontal inflammation [25,26]. In addition, inflammatory cytokines trigger receptor activator nuclear kappa β ligand (RANK-L) expressions on osteoblasts and T helper cells. RANK-L on osteoblasts and T helper cells then interacts with RANK on osteoclast precursors. Mature osteoclasts mediate alveolar bone destruction [27].

In summary, the microbial biofilm is the primary factor in the pathogenesis of the periodontitis; however, this biofilm alone is not sufficient for the onset of a disease. As included in the 2017 classification, systemic diseases or conditions can dominate risk factors and affect the host’s response to microbial plaque. It is known that factors such as smoking [28], mouth breathing [29], diabetes [30], stress [31], and atherosclerosis [32] are effective in the formation and progression of periodontal disease. On the contrary, diabetes, rheumatoid arthritis, and cardiovascular diseases have been considered a risk factor for periodontitis. Strikingly, the relationship between neurodegenerative diseases and periodontitis has also been reported as bidirectional. In a very recent study, the authors reported that Parkinson’s disease increases inflammation in periodontal tissues and that this increase is associated with neuroinflammation [33]. Based on the evidence in the literature, it should be noted that identifying and/or modifying these factors in the control and treatment of periodontitis will support to prevent this disease and implement an appropriate treatment protocol.

## 4. Human Beta-Defensins in Periodontal Tissues

Oral mucosal colonization starts at birth. Beta-defensins, which are already expressed during the development of an embryo, play a significant role in the characterization of maternally derived oral microbiomes [34]. The oral cavity is a warm, moisty, and nutrient-rich environment, which allows for the colonization and growth of a wide range of microorganisms, including bacteria, viruses, fungi, archaea, and protozoa [35]. In a healthy oral cavity, interactions between oral mucosal tissues and microbes are synergistic. Commensal or opportunistic-to-commensal microbes of the oral cavity maintain a healthy digestive tract, present anti-oxidative activity, and contribute to wound healing and angiogenesis through the maintenance of nitric oxide homeostasis and even suppress the growth of pathogens through the activation of antimicrobial peptide expressions [36,37]. Resident cells of oral mucosal tissues, together with neutrophils, are strong producers of various host defense peptides such as calprotectin, hCAP18/LL-37, human neutrophilic alpha defensins (HNPs), and hBDs. Among these, hBDs are a group of host defense peptides that are widely expressed by kerationyctes, fibroblasts, and monocytes. These cationic peptides are formed of a β-sheet structure and three disulfide bonds and have a size of 2–5 kDa [38]. The genes encoding HNPs and hBDs are located within chromosome region 8p21–p23. Although more than 30 hBD genes were identified in the human genome, only 4 hBDs, hBD-1, -2, -3, and -4, were found to be expressed and secreted in human gingivae [39].

In the oral cavity hBDs can be detected in soft tissues (e.g., gingiva, tongue, and oral mucosa) and in oral fluids (i.e., saliva and gingival crevicular fluid). hBD expressions and secretions are regulated at the transcriptional level. With this, hBDs differ from HNPs, as the secretion of the latter is regulated post-translationally [5]. hBD-1 is constitutively expressed by human oral mucosal epithelial cells, while the expression of hBD-2 and hBD-3 require the presence of infectious or inflammatory stimulants [40]. The inducible characters of hBD-2 and hBD-3 are related to the regulatory elements (NF-κB for hBD-2 and GAS for hBD-3) in their promoter regions [41]. Interestingly, the expression of hBDs in the gingiva is not solely dependent on microbial or inflammatory activation, but keratinocyte differentiation also plays a significant role in it [42]. While hBDs are widely observed in various epithelial layers of the oral and sulcular epithelium, they are not expressed in the undifferentiated junctional epithelium, where constant bacterial challenges and inflammatory response take place. A rather high HNP expression by neutrophils is observed in the junctional epithelium [43]. Furthermore, hBD-1 and hBD-2 are expressed in the granular and spinous layers of the stratified epithelium, in contrast to hBD-3, which is expressed in the basal epithelium. These findings indicate that the expression profiles of gingival antimicrobial hBDs have region-specific characteristics.

**Figure 1:**
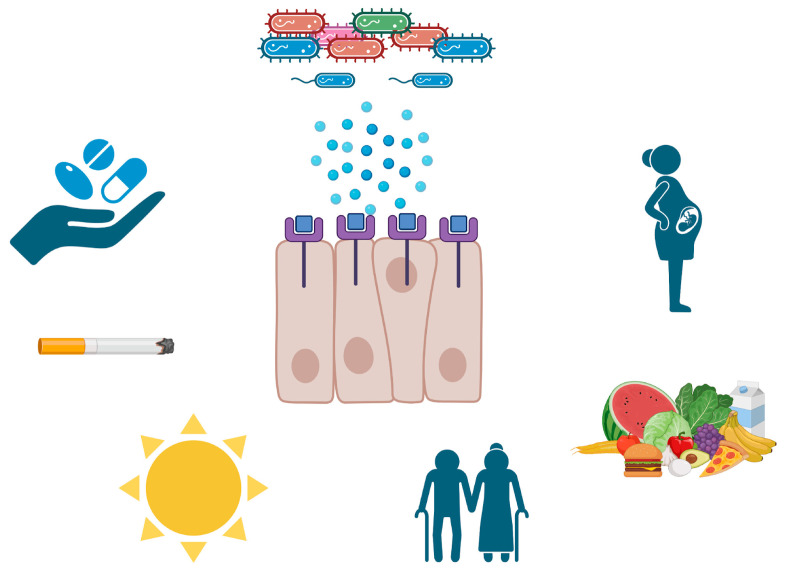
Lifestyle, behavioral, local, and environmental factors influence intraoral human beta-defensin expression and secretion profiles. Key influences include hormonal changes, diet, smoking, aging, nutrition, and environmental exposures.

Although all hBDs demonstrate antimicrobial activities, hBD-3 is the most potent one. Additionally, hBDs can recruit T- cells, mast cells, and neutrophils and can stimulate epithelial cell differentiation and migration, linking innate and adaptive responses with wound healing [44]. On the one hand, hBD levels are expected to increase in patients with periodontal diseases, as the infectious stress and inflammatory response increase during these diseases [45]. On the other hand, various attempts to relate oral hBD levels to the severity or extension of periodontal diseases ended up with conflicting results; hBD levels were found to be increased, decreased, or unchanged during the development of periodontal diseases [3]. As previously mentioned, hBD expression is dependent on microbial and inflammatory stimuli; however, hBDs are also prone to enzymatic degradation by bacterial and human proteases [46]. This suggests that an increase in the microbial mass may elevate and suppress hBD levels simultaneously. Furthermore, hBD expression is not only regulated by the genes encoding β-defensins but is also regulated by the genes encoding eukaryotic pattern-recognition receptors [47]. Toll-like receptors (TLRs) are eukaryotic pattern-recognition receptors that are specialized to recognize microbe-associated molecular patterns. Previous studies demonstrated that the single-nucleotide polymorphisms of TLRs are associated with an increased colonization of periodontitis-associated bacteria [48], a risk of developing periodontitis [49], and decreased hBD levels [47]. Finally, one’s lifestyle and behaviors and systemic diseases and conditions can stimulate or inhibit intraoral hBD expression and secretion profiles (Figure 1).

## 5. Systemic Factors That Affect Both hBDs and Periodontal Tissues

### 5.1. Hyperglycemia

Hyperglycemia, the main pathological hallmark of diabetes mellitus, markedly interferes with homeostatic epithelial integrity, leading to an abnormal influx of immune-stimulatory microbial products [50,51]. Indeed, hyperglycemia impairs the expression of intercellular adhesion molecules and increases intercellular permeability in gingival epithelial cells and leads to hyperglycemia-related AGE (Advanced Glycation End product) signaling, oxidative stress, and ERK1/2 activation [52]. The effect of a high-glucose environment on oral hBD levels has been the subject of several in vitro studies. The results reveal that the release of hBD1 increases in proportion to the glucose level in the environment; however, the underlying mechanism has yet to be elucidated [53,54]. The effects of hyperglycemic conditions on hBD2 and hBD3 expressions, on the other hand, differs from hBD-1. It was found that keratinocytes, which are exposed to a high-glucose environment, reduce hBD2 and hBD3 expressions due to the downregulation of STAT-1 (Signal Transducer and Activator of Transcription 1) and the inhibition of p38MAPK (p38 Mitogen-activated Protein Kinase) signaling, respectively. On both occasions, increased AGE formation in keratinocytes plays a critical role [55].

It is also worth mentioning that hBDs can regulate glycose levels as well. In an animal study, where the impact of hBDs on hyperglycemic conditions was investigated, recombinant hBD2 was found to reduce glucose levels, which typically increase with high-fructose and Western-style diet (WSD). These findings suggest that the relation between hyperglycemia and hBD2 can be reciprocal, and chronic hyperglycemia may disturb the hBD2 regulation of blood glucose levels [56]. Several animal and clinical studies examining the effects of hyperglycemic conditions on hBDs have utilized diabetic participants as their study group [57,58,59,60,61]. Diabetes is a complex metabolic syndrome characterized by the release of AGEs as a result of uncontrolled and chronic hyperglycemia, and its pathogenesis is highly affected by various predisposing factors of a genetic and lifestyle nature [62]. Thus, the expression profile of diabetes-regulated host defense peptides is not included in this review. A general summary of research studies on alterations in beta-defensin release under hyperglycemic conditions is presented in Table 1.

### 5.2. Retinoic Acid Use

Vitamin A, through its active metabolite retinoic acid, acts in various biological conditions such as embryonic development, hormone function, the maintenance and regulation of immune responses, and the homeostasis of the epithelium and mucosa [65,66]. Retinoids mediate a broad range of effects on the immune system, including the development of primary and secondary lymphoid tissues, the functional differentiation of various immune cells, and the control of inflammatory responses [67].

There are only a limited number of studies in the literature that evaluate the keratinocyte hBD response in relation to retinoic acid stimulation. An in vitro study using skin keratinocytes revealed that pro-inflammatory cytokines and *P. aeruginosa*-induced hBD-2, -3, and -4 gene expressions were reduced by retinoic acid treatment [68]. Another in vivo study showed that topical retinoic acid increased mice BD-3 levels and mRNA expressions [69].

13-cistrans-retinoic acid, or isotretinoin, is a molecule that occurs naturally in the body as a result of the vitamin A metabolism. Isotretinoin is a first-generation synthetic vitamin A analogue used in the treatment of many dermatological diseases, especially acne vulgaris [70]. In various studies examining skin biopsies from patients with acne vulgaris who received systemic isotretinoin, it has been demonstrated that retinoic acid does not affect cutaneous hBD-1 levels, but it does reduce cutaneous hBD-2 levels [71,72].

In our team’s clinical study, which is the first of its kind in the literature, we aimed to investigate the impact of systemic retinoic acid use on oral hBD levels. Our findings reveal that systemic retinoic acid use was associated with suppressed salivary hBD-2 levels. Furthermore, our research showed that systemic retinoic acid use had no effect on the hBD levels of the serum and gingival tissue [73]. Retinoic acids’ precise mechanism of action in relation to beta-defensin releases remains unclear; however, it has been hypothesized that AP-1 antagonization [68] and Treg cell activation may be key factors in this process [73]. A general summary of research studies on alterations in beta-defensin release with retinoic acid use is presented in Table 2.

### 5.3. Vitamin D Use

Vitamin D is essential for health and disease prevention. While the primary function of vitamin D is to regulate mineral and skeletal homeostasis in the body, there is a wealth of literature that highlights its pleiotropic effects. These effects include immune modulation, an improvement of endothelial and mucosal functions, and a regulation of the glucose metabolism [74]. Vitamin D may improve the immune defense against infections by a modulation of the innate immune function [75].

Studies have produced inconsistent results regarding the impact of vitamin D use on hBD release. Additionally, there is no clarity in the literature regarding the mechanism of action of vitamin D on hBDs. The induction of CYP27B1 and CYP27A1, regulation of NOD2 expression, and activation of the IL-22 signaling pathway are mentioned as functioning pathways to explain vitamin D-induced hBD regulations [76,77,78,79]. Finally, it is also stated that the stimulation of hBD expressions by vitamin D requires IL-1β and TLR activation [80].

Meanwhile, vitamin D3 supplementation at a dose of 10 µg/day does not appear to affect DEFB4 gene expressions in the oral mucosa of healthy children [81]. Topical vitamin D3 applications have been reported to cause a decrease in hBD-2 expressions in psoriasis patients. It has been suggested that topical treatment may indirectly reduce hBD-2 expressions by affecting the amount of pro-inflammatory cytokines and Ca2 [82]. An in vitro study investigated the connection between human HBDs and immune infiltration in periodontitis to determine whether vitamin D3 plays a regulatory role. The results show that vitamin D3 upregulated the expression of HBD-2 and HBD-3 in human gingival fibroblasts through the CYP27A1 enzyme. Additionally, vitamin D3 was found to affect the TNF signaling pathway or the NF-κB signaling pathway. Thus, vitamin D3 diminished the increase in HBD-2 and HBD-3 induced by the TNF-α/*P.gingivalis*-LPS in hGFs [77]. A similar outcome was discovered in a study exploring the consequences of vitamin D on the growth and adhesion of *P. gingivalis* to the human gingival epithelium and periodontal ligament cells, as well as its immunomodulatory effect on HBD-3 and other cytokines. Filippis et al. found that vitamin D has the ability to reduce the levels of pro-inflammatory cytokines, while promoting the production of HBD-3 [83].

Animal studies indicated that the local application of vitamin D increases endogenous BD levels in cows with active digital dermatitis lesions, characterized by ulcers and impaired wound healing with *Treponema* species. Indeed, it has been hypothesized that this effect of vitamin D is achieved through TLR-2 activation [84]. In addition, it has been reported that the release of hBD-1, -2, and -3 and IL-21 is suppressed in cyp2r1 mutant zebrafish lacking the capacity to metabolize vitamin D and vitamin D-induced zf-BD expressions in zebrafish intestines by activating IL-22 signaling [78].

In conclusion, while there is evidence to claim that vitamin D may stimulate hBD expressions, the use of vitamin D supplements to boost hBD expressions is still far from routine daily practices [85]. A general summary of research studies on alterations in beta-defensin release with vitamin D use is presented in Table 3.

### 5.4. Pregnancy

Pregnant women constitute a unique population group due to their heightened susceptibility to certain infectious diseases, which is attributed to their immunological state [87]. A bacterial infection of the uterus can lead to pregnancy complications, including preterm labor, neonatal infection, and postpartum endometritis. Moreover, viral infections, such as HIV, herpes simplex, and human papilloma virus, can risk the health of both the mother and the baby. The role of host defense peptides in preventing such complications is suggested by their expression and regulation during pregnancy [88].

The female reproductive tract (FRT) is significantly influenced by the hypothalamic–pituitary–ovarian axis, which affects both the innate and adaptive immune responses in the FRT [89]. HBD levels in the FRT vary not only throughout the menstrual cycle but also during pregnancy. The hormonal regulation of FRT hBDs is mainly associated with the varying expressions of pattern-recognition receptors (PRRs), such as toll-like receptors (TLRs), during the menstrual cycle [90].

HBD-1, -2, and -3 are widely expressed in a pregnant uterus, with expressions detected in the amnion, decidua, chorion, and placental trophoblasts [91,92,93,94,95]. HBD-1–3 peptides have also been identified in amniotic fluid and human vernix [96]. HBD levels have been reported to differ across the three trimesters of a pregnancy [97]. HBD-1 concentrations in amniotic fluid vary with the gestational age and are higher in the second trimester [98]. Also, HBD-3 increases during the process of a labor term and also increases in women with spontaneous preterm labor with intact membranes or a preterm prelabor rupture of membranes with intra-amniotic inflammation or intra-amniotic infections [99]. Under circumstances of pregnancy disorders, abnormal levels of defensins were found to be associated with specific situations. The concentration of hBD-2 in the amniotic fluid in the second trimester was associated with the premature rupture of membranes but not with a preterm birth [100].

The number of human studies investigating the changes in oral beta-defensin expressions during pregnancy is limited. Research findings indicate that the levels of hBD-1 and hBD-2 in the saliva of pregnant women fluctuate and decrease significantly, particularly during the third trimester. In contrast, the salivary levels of hBD-3 remain constant throughout pregnancy and the postpartum period [101]. However, one study did not observe any significant difference in salivary hBD-1 levels between pregnancy and postpartum [102]. Considering that the number of studies that evaluate the effects of pregnancy on oral hBD levels is highly scarce, there is a definite need for more extensive clinical studies. A general summary of research studies on alterations in beta-defensin release with pregnancies is presented in Table 4.

### 5.5. Aging

Evidence indicates that certain aspects of the immune function may undergo alterations as individuals age, thereby increasing their vulnerability to infections. With aging, modifications occur in both the adaptive and innate immunity; however, this does not necessarily result in immunodeficiency but rather the dysregulation of immune responses [103,104]. The exact mechanism of aging on hBD release is not yet entirely clear, although some hypotheses are presented. It has been hypothesized that the diminution of the oral epithelium’s density and regenerative capacity as a consequence of aging could have an impact on the expression of hBDs [105,106]. Moreover, with aging, a reduction in the density of dentric cells, which play a critical role in the release of hBDs, may also have an effect [107].

Few studies have explored the oral hBD profiles of elderly patients. In an in vitro study, hBD-1, -2, and -3 expressions were observed to increase with age [108], which contrasts with the decreased salivary hBD-2 levels found in elderly individuals compared to young ones [107]. Another study found that serum hBD-2 levels did not change with age [109]. However, the detection of age-related differences in the localization of hBD-2 in the human gingiva suggests that oral and systemic hBD expressions may be regulated differently [110]. Finally, a comparison of salivary hBD-1–3 levels between aged (>65 years) [106] and working-aged (40–60 years) [47] groups suggests no difference in salivary hBD levels in relation to aging. A general summary of research studies on alterations in beta-defensin with aging is presented in Table 5.

## 6. Limitations and Future Prospects

Today, the contribution of lifestyle and systemic conditions on immune responses is widely accepted; however, there is still lack of information on how these modifiable factors can regulate oral host defense peptide, especially hBD, expressions. A majority of the evidence comes from small-scaled cross-sectional studies, which do not allow for a description of cause–effect relationships. Indeed, it is also true that due to ethical reasons, systemic conditions and their effects on hBD expressions cannot be followed without being treated.

The research studies reviewed in this article, which explore the factors that affect beta-defensin release, exhibit diverse methodologies and are not repeated studies. The heterogeneous composition of these articles generates a mass of valuable but unverified data. Despite the challenges posed by the data we have, current studies illuminate potential avenues for future studies. However, following the changes in oral hBD expressions in individuals who participate in diet-regulation programs or who receive vitamin or mineral supplements under the control of a physician will help researchers to profile the role of environmental factors on host defense peptides.

## 7. Summary and Conclusions

Current evidence does not support the fact that hyperglycemia, long-term vitamin use, pregnancy, or aging regulate the periodontal status via their effects on oral hBD expressions. However, there is also strong evidence that hyperglycemia, which is associated with being overweight and obesity and diabetes, is related to a diminished immune response, including an impaired oral hBD expression. Thus, omitting excessive sugar consumption can be advised to maintain a strong periodontal immune response. Finally, in vitro studies hint at the fact that vitamin D may stimulate hBD expressions, which might be beneficial, especially for individuals with suppressed immune responses.

## Figures and Tables

**Table 1:** Summary of studies evaluating the hBD levels in hyperglycemia.

Author, Year	Population	Condition	Defensin Type	Detection Method	Findings
Malik et al. 2006 [54]	Human mesangial cells.	Hyperglycemia	HBD-2	Quantitative real-time PCR.	hBD-1 expression increases to correct increased oxidative stress resulting from hyperglycemia.
Barnea et al. 2008 [53]	Human embryonic kidney and colon adenocarcinoma cells.	Hyperglycemia	HBD-1	Quantitative real-time PCR.	High concentrations of glucose enhanced hBD-1 expression, and these levels were further elevated after insulin treatment.
Lan et al. 2011 [55]	Keratinocyte cultures.	Hyperglycemia	HBD-2	Real-time quantitative PCR, and small interfering RNA, Western blot.	A high-glucose environment reduces hBD2 expression of keratinocytes via downregulation of STAT-1 signaling.
Diaz et al. 2014 [63]	Human neonate keratinocytes.	Hyperglycemia	DEFB-1	Q-PCR and cell culture.	At 24 h of glucose exposure (45.5 mM), DEFB1 expression was significantly upregulated.
Kiselar et al. 2015 [64]	Recombinant hBD-2.	Hyperglycemia	HBD-2	Proteolysis and MS analysis, radial diffusion assay, and chemotaxis assay.	HBD-2 is susceptible to altering modifications of Arg and Gly residues by dicarbonyl molecular species.
Rosa et al. 2023 [56]	84 mice that received HD51–9 (n = 28), hBD2 (n = 28), and BSA (n = 28).	Hyperglycemia	HBD-2	Oral glucose tolerance test, immunohistochemical analysis, organoid cell culture, and real-time PCR.	Administration of either HD51–9- or hBD2-attenuated glucose intolerance, resulting in a lowering of blood glucose.

**Table 2:** Summary of studies evaluating the hBD levels with retinoic acid use.

Author, Year	Population	Condition	Defensin Type	Detection Method	Findings
Harder et al. 2004 [68]	Keratinocytes.	Retinoic acid use	HBD-1, -2, -3, and -4	Real-time RT-PCR, Luciferase gene reporter assay, and Western blot.	Treatment of keratinocytes with ATRA downregulated *P. aeruginosa*-mediated hBD-2, -3, and -4 gene expressions.
Lee et al. 2010 [69]	Hairless mice (no additive data).	Retinoic acid use	mBD3	Immunostaining and RT-PCR.	Topical retinoids upregulate mBD3 RNA expression in mouse skin.
Aksoy G. et al. 2018 [72]	Acne vulgaris patients (n = 44) and a control (n = 20).	Retinoic acid use	HBD-2	Immunohistochemical staining.	Systemic use of isotretinoin decreased cutaneous hBD-2 levels, with no difference in hBD-1 levels.
Atalay et al. 2022 [73]	Systemic retinoic acid users (n = 34) and a control (n = 35).	Retinoic acid use	HBD-1, -2, and -3	ELISA and immunohistochemical staining.	Systemic retinoic acid use was associated with a suppressed salivary hBD-2 level, which was independent of gingival inflammation.

**Table 3:** Summary of studies evaluating hBD levels in vitamin D use.

Author, Year	Population	Condition	Defensin Type	Detection Method	Findings
Van Der Velden et al. 2009 [82]	Psoriasis patients (n = 6).	Vitamin D use	HBD-2	Immunohistochemical staining.	HBD2 expressions decrease in psoriasis lesions after topical vitamin D treatment.
Wang et al. 2010 [79]	Primary human monocytic and epithelial cells.	Vitamin D use	HBD-2 and -3	Western blotting and RT/qPCR.	Regulation of NOD2 expression by vitamin D affects the downregulation of HBD release.
Klug-Micu et al. 2013 [76]	Monocytes.	Vitamin D use	DEFB4	Radioimmunoassay, PCR, flow cytometry, PBMC assays, and T-cell clone assays.	CD40L and IFN-γ can activate the vitamin D-dependent antimicrobial pathway by inducing CYP27B1, VDR, cathelicidin, and DEFB4.
Merriman et al. 2015 [86]	Lactating cows’ monocytes, neutrophils, and epithelial cells.	Vitamin D use	Bovine β-defensin-3, -4, -6, -7, and -10	Q-PCR.	Multiple β-defensin genes are upregulated by 1,25-dihydroxyvitamin D3 in cattle.
Han et al. 2017 [85]	Patients with respiratory failure (n = 30).	Vitamin D use	HBD-2	ELISA, IDS-İsys, and RT-qPCR.	There were no correlations between changes in total and free 25 (OH)D and hBD-2 concentrations.
Watts et al. 2019 [84]	Cows with digital dermatitis (n = 15).	Vitamin D use	β-defensin tracheal antimicrobial peptide (TAP)	Q-PCR.	Vitamin D3 can boost endogenous β-defensins in cow skin.
Liao et al. 2023 [78]	Cyp2r1 mutant zebrafish.	Vitamin D use	zfBD-1, -2, and -3	qRT-PCR, in vivo luciferase assay, and Western blotting.	The expressions of zfBD-1, -2, and -3 were reduced in VD-deficient zebrafish. VD induced zfBD expression in zebrafish intestines by activating IL-22 signaling.
Zhang et al. 2023 [77]	Human gingival fibroblasts.	Vitamin D use	HBD-2 and -3	RT-PCR.	Vitamin D3 upregulated the expression of HBD-2 and HBD-3 through CYP27A1.

**Table 4:** Summary of studies evaluating hBD levels in pregnancies.

Author, Year	Population	Condition	Defensin Type	Detection Method	Findings
King et al. 2007 [95]	Placental and chorion trophoblasts.	Pregnancy	HBD-1, -2, and -3	RT-PCR and immunohistochemical analysis.	Fetal membranes and placenta are key sources of HBDs in the uterus at term. The expression of selective HBDs can be upregulated in these tissues in response to inflammatory cytokines.
Gürsoy et al. 2016 [101]	Pregnant (n = 30) and non-pregnant women (n = 24).	Pregnancy	HBD-1, -2, and -3	ELISA.	Pregnancy has suppressive effects of salivary concentrations of hBD-1 and hBD-2, while that of hBD-3 remains unaffected.
Lasisi et al. 2018 [102]	Pregnant women (n = 47).	Pregnancy	HBD-1	ELISA.	Salivary HBD-1 did not show a significant difference when comparing levels during pregnancy and postpartum.

**Table 5:** Summary of studies evaluating hBD levels with aging.

Author, Year	Population	Condition	Defensin Type	Detection Method	Findings
Matsuzaka et al. 2006 [110]	Young group (n = 6) and elderly group (n = 7).	Aging	HBD-2	Immunohistochemistry staining.	The results reveal HBD-2-positive cells in spinous cells in the elderly group and in the parakeratinized layer in the young group.
Delgado et al. 2013 [109]	Mononuclear cells of participants from 25 to 34 years old (n = 23) and participants from 65 to 84 years old (n = 21).	Aging	HBD-2	ELISA and RT-qPCR.	HBD-2 production in serum is not affected by aging.
Shimizu et al. 2016 [107]	Participants from 65 to 85 years old (n = 168) and from 22 to 30 years old (n = 26).	Aging	HBD-2	ELISA.	Elderly participants had lower salivary hBD-2 secretion levels than young participants.
Gilbert et al. 2021 [108]	Human epithelial cells of SARS-CoV-2-negative, asymptomatic SARS-CoV-2-positive, and symptomatic SARS-CoV-2-positive patients with different age groups.	Aging	HBD-1, -2, and -3	RT-qPCR.	hBD-1–3 mRNA transcript levels and age were correlated.

## Data Availability

No new data were created or analyzed in this study.
